# Development of a written assessment for a national interprofessional cardiotocography education program

**DOI:** 10.1186/s12909-017-0915-2

**Published:** 2017-05-18

**Authors:** Line Thellesen, Thomas Bergholt, Morten Hedegaard, Nina Palmgren Colov, Karl Bang Christensen, Kristine Sylvan Andersen, Jette Led Sorensen

**Affiliations:** 1 Department of Obstetrics, The Juliane Marie Centre for Children, Women and Reproduction, Rigshospitalet, University of Copenhagen, Blegdamsvej 9, DK-2100 Copenhagen, Denmark; 2 Section of Biostatistics, Department of Public Health, University of Copenhagen, Oester Farimagsgade 5, Building 15.2.12, DK-1014 Copenhagen, Denmark

**Keywords:** Cardiotocography, Fetal monitoring, Written assessment, Multiple-choice question, Validity, Interprofessional, Continuing professional development

## Abstract

**Background:**

To reduce the incidence of hypoxic brain injuries among newborns a national cardiotocography (CTG) education program was implemented in Denmark. A multiple-choice question test was integrated as part of the program. The aim of this article was to describe and discuss the test development process and to introduce a feasible method for written test development in general.

**Methods:**

The test development was based on the unitary approach to validity. The process involved national consensus on learning objectives, standardized item writing, pilot testing, sensitivity analyses, standard setting and evaluation of psychometric properties using Item Response Theory models. Test responses and feedback from midwives, specialists and residents in obstetrics and gynecology, and medical and midwifery students were used in the process (proofreaders *n* = 6, pilot test participants *n* = 118, CTG course participants *n* = 1679).

**Results:**

The final test included 30 items and the passing score was established at 25 correct answers. All items fitted a loglinear Rasch model and the test was able to discriminate levels of competence. Seven items revealed differential item functioning in relation to profession and geographical regions, which means the test is not suitable for measuring differences between midwives and physicians or differences across regions. In the setting of pilot testing Cronbach’s alpha equaled 0.79, whereas Cronbach’s alpha equaled 0.63 in the setting of the CTG education program. This indicates a need for more items and items with a higher degree of difficulty in the test, and illuminates the importance of context when discussing validity.

**Conclusions:**

Test development is a complex and time-consuming process. The unitary approach to validity was a useful and applicable tool for development of a CTG written assessment. The process and findings supported our proposed interpretation of the assessment as measuring CTG knowledge and interpretive skills. However, for the test to function as a high-stake assessment a higher reliability is required.

**Electronic supplementary material:**

The online version of this article (doi:10.1186/s12909-017-0915-2) contains supplementary material, which is available to authorized users.

## Background

Cardiotocography (CTG) is a widely used fetal surveillance method. Errors in the management of CTG are a recognized cause of adverse obstetric outcomes [1, 2]. Omission of use when indicated, misinterpretation, and delay in action are some of the described errors that can lead to severe fetal neurological damage or death. Regular education and training in fetal surveillance to all staff responsible for laboring women is recommended [3].

In 2012, a comprehensive national obstetric intervention (*Safe Deliveries*) was initiated in Denmark with the aim of increasing the quality of patient care and reducing hypoxia among newborns [4]. The Danish Regions, the Danish Society of Obstetrics and Gynecology, the Danish Association of Midwives, the Danish Pediatric Society, the Danish Society for Patient Safety and the Patient Compensation Association all supported the initiative. As part of the intervention all midwives and physicians working at a maternity unit in Denmark had to complete a CTG education program, consisting of an e-learning program, a one-day course, and a final written assessment.

CTG training leads to improved interpretive skills, better management of intrapartum CTG, and higher quality of care, but a lack of validated assessment methods has been indicated [5]. Comprehensive fetal surveillance education and credentialing programs exist in the United States, in Australia and New Zealand [6, 7], and an intervention similar to *Safe Deliveries* was implemented in Sweden in 2007 [8]. To ensure coherence to national guidelines and context a separate Danish CTG education and assessment program was developed.

Validity is known to be the single most important factor when discussing assessment, and all assessments require evidence of validity [9]. Validity refers to the evidence presented to support or refute the proposed interpretations of the assessment. Thus, validity can be seen as an argument for the interpretations. Validity is not a definite size but always a matter of degree, neither is it a property of the instrument (in this case the written assessment) but of the interpretations made upon the instrument’s score [9]. Reliability is a necessary component of validity that refers to the reproducibility and consistency of the scores of the assessment [10].

We chose the multiple-choice question (MCQ) format for the assessment in the CTG educational program. In addition to validity and reliability, educational impact, cost effectiveness and acceptability needs to be taken into account in the process of test development [11]. MCQ testing is time- and cost effective and suitable for large groups.

The aim of this article was to describe and discuss the process of developing a CTG MCQ test to be used in a national CTG education program, and to introduce a feasible and acknowledged method for written test development in general. In the process we collected evidence to support or refute the proposed interpretation that the assessment measured knowledge, interpretive skills, and clinical decision-making concerning fetal surveillance with CTG.

## Methods

### Setting and context

**Fig. 1:**
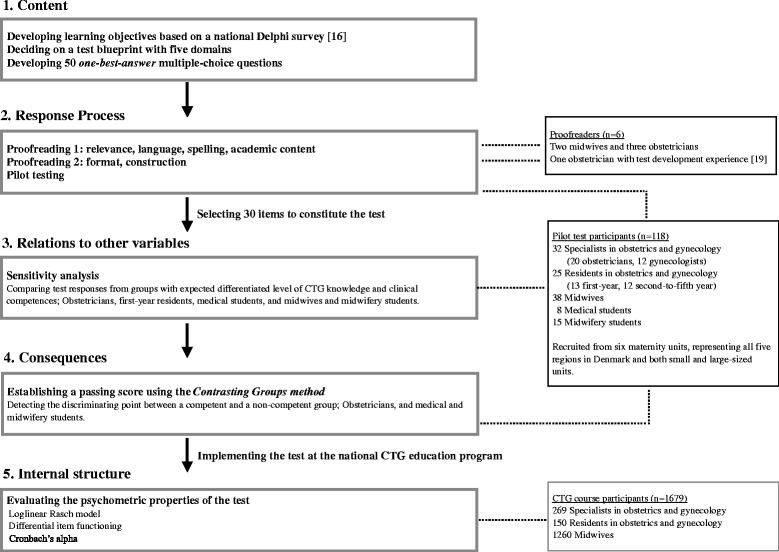
Study design. Flowchart of the five sources of validity evidence and the participants involved

Data collection took place from December 2012 to December 2013. The Danish maternity units (*n* = 24) were distributed among five regions and numbers of annual deliveries ranged from 235 to 6555 [12]. In this study, physicians refer to specialists and residents in obstetrics and gynecology. In Denmark, specialists work mainly within obstetrics (obstetricians), gynecology (gynecologists) or, in smaller units, within both fields. Residency extends over five years and consists of first-year residency followed by second-to-fifth-year residency. The included participants are presented in Fig. 1.

### Five sources of validity evidence

In the present study, we perceive validity as a unitary concept, with construct validity as the overall term [13]. Construct validity refers to what the test is proposed to measure. Evidence to support validity was collected from five sources based on *The Standards for Educational and Psychological Testing* [14]: content, response process, relations to other variables, consequences, and internal structure, which will be described in detail in the following. The study design is illustrated in Fig. 1.

### Content (do the items represent the construct?)

Learning objectives: Learning objectives are essential when developing an educational intervention, as they define what learners should know and master after the intervention [15]. We developed objectives based on national consensus amongst midwives and obstetricians in a national Delphi study [16]. The content of an assessment should always represent the most important subjects, therefore, objectives with the highest relevance rating constituted the content of the test.

Blueprint: Also based on the rated objectives we decided on a five-domain test blueprint: fetal physiology (24%), indication (3%), equipment (3%), classification (33%) and management (37%). A blueprint is a framework that describes the subcategories (domains) in the test and specifies the proportion of items in each subcategory [9].

**Fig. 2:**
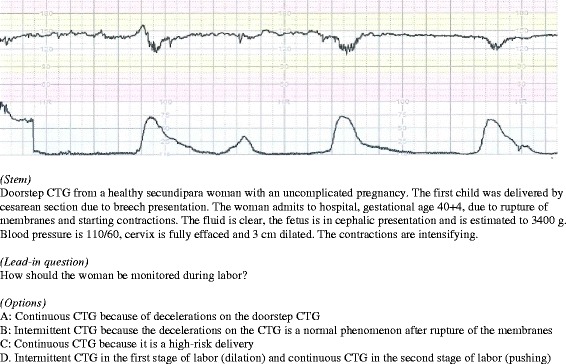
Example of a multiple-choice question in a *one-best-answer* format

MCQ: The MCQ’s were constructed in a *one-best-answer* format [17–19]. The items consisted of a stem (predominantly a clinical case scenario) and a lead-in question, followed by a series of three or four options. The literature suggests that three options are adequate, but a fourth can be applied when plausible [20]. We emphasized to develop items that required problem solving and clinical reflection and not just recall of knowledge. An obstetrician with profound experience in CTG teaching and clinical use of CTG (NPC) constructed the first draft of items in collaboration with two members of the research group (LT and KSA). An item example is illustrated in Fig. 2.

The entire CTG test can be obtained from the corresponding author at the request of units or organizations who wish to use the test.

### Response process (are the thought processes of the test-takers related to the intended construct?)

Proofread: The items were initially evaluated in two rounds of proofreading, in which three of the proofreaders (MH, TB, JLS) were members of the research group (Fig. 1). In the first proofreading, item relevance, language, spelling, and academic content were critically reviewed and in the second proofreading, item format and construction.

Pilot test: The items were subsequently evaluated in a pilot test, in which the participants represented the intended test-takers; midwives, and specialists and residents in obstetrics and gynecology from all five regions of Denmark (Fig. 1). Medical and midwifery students were additionally included in the pilot testing to examine the test’s discrimination abilities. The pilot participants were asked to answer and comment on the test and time for test completion was measured. The pilot testing was conducted during visits to the relevant maternity units and midwifery school. A member of the research team was present during the testing, which allowed both written and verbal feedback, ensured individual test responses, and secured test confidentiality.

During the response process the research group iteratively revised items and excluded non-functioning items. At the end of the response process the research group decided which items to implement in the test.

### Relations to other variables (are test responses correlated with scores from a similar instrument?)

No other CTG test was available to relate to the current test. Therefore, we related the test to level of clinical competences and compared test responses from groups with expected differentiated level of CTG knowledge and clinical competences within each profession. Among physicians we compared test responses from obstetricians, first-year residents and medical students. Among midwives we compared test responses from midwives and midwifery students. Test responses from pilot participants were used in this sensitivity analysis.

### Consequences (how is the passing score determined? What are the consequences for the test-takers? Are patient outcomes improved?)

We established a criterion-based passing score for the CTG test using the *Contrasting Groups method*. This method defines the passing score as the best discriminating point between a competent group and a non-competent group [21]. We defined obstetricians as competent and medical and midwifery students as non-competent. We chose obstetricians as competent as they represent a defined group with at least five years of clinical obstetric experience. The group of midwives consisted of both newly educated and experienced midwives, thus this group was more heterogeneous. Test responses from pilot participants were used. The consequences of a participant’s test results were a local decision taken between the participant and the clinical director in each maternity unit. Repeated participation in the CTG course and test was possible. A possible improvement in patient outcome will be evaluated in a separate study.

### Internal structure (are the psychometric properties acceptable?)

We examined the test’s psychometric properties using the test responses from the participants at the national CTG courses (Fig. 1). The analyses are described in the statistics and in Additional file 1.

### Statistics

Test sensitivity was measured using a Mann-Whitney test. *P*-values < 0.05 were considered statistical significant.

The loglinear Rasch model was used to examine the fit of each item. This Item Response Model integrates both the ability of the test-taker and the difficulty of the item when measuring the probability of a correct answer [22]. Examination of model fit can provide information about how justified it is to measure the construct with the chosen items [23].

Differential item functioning (DIF) was evaluated concerning profession, geographical regions, seniority, and size of maternity unit. DIF arises when an item performs differently in various subgroups [24].

The analyses were adjusted for multiple testing using the Benjamini and Hochberg procedure [25]. *P*-values < 0.05 were required for statistical significance.

Cronbach’s alpha was calculated as an estimate for reliability both in the context of pilot testing and in the context of the CTG education program. A Cronbach’s alpha value above 0.7 is regarded as acceptable, whereas a value above 0.9 is required for high-stake and certification assessments, in which the results can have serious impact on an examinee [9, 24].

Data were entered using double-entry technique. Statistical analyses were performed using SAS version 9.4 (SAS Institute Inc., Cary, NC, USA) and the DIGRAM software package (Department of Biostatistics, University of Copenhagen, Denmark). Supplementary details on the psychometric properties and the statistical aspects of validation are outlined in Additional file 1.

## Results

We initially developed 50 items for the national CTG test. Three items were excluded during proofreading and six items during pilot testing. Items were excluded due to similarity, extensive stem text, imprecise response options, different construct than intended, and lack of evidence in relation to item content. We selected 30 items to constitute the test based on the blueprint, the comments and responses from the pilot test participants and the time devoted for completion of the test at the national CTG course. Several items concerning management showed not to function optimally, which meant the initial blueprint could not be completely adhered to. The blueprint was distributed as follows: fetal physiology (27%), indication (7%), equipment (3%), classification (33%), and management (30%). Proportion of correct answers in the 30-item test among the pilot test participants is presented in Table 1. Cronbach’s alpha equaled 0.79.

**Table 1:** Psychometric properties. Proportion of correct answers, loglinear Rasch model fit, and differential item functioning (DIF) in the 30-item CTG test

Item	Blueprint domain	Pilot test participants Proportion of correct answers in percent *n* = 118	CTG course participants Proportion of correct answers in percent *n* = 1679	Loglinear Rasch			DIF
				Observed	Expected	*P*-value	*P*-value
Item1	Indication	81.4	97.7	0.350	0.346	-	*
Item2	Classification	78.8	91.8	0.737	0.685	-	-
Item3	Classification	82.2	92.9	0.795	0.751	-	-
Item4	Classification	80.5	97.0	0.524	0.530	-	-
Item5	Equipment	94.1	99.3	0.134	0.348	-	-
Item6	Management	94.1	99.5	0.537	0.348	-	-
Item7	Indication	74.6	93.9	0.466	0.372	-	*
Item8	Classification	73.3	89.7	0.296	0.341	-	*
Item9	Classification	57.6	70.0	0.153	0.242	-	-
Item10	Management	86.4	92.1	0.278	0.342	-	-
Item11	Physiology	72.9	95.6	0.371	0.345	-	-
Item12	Physiology	80.5	96.7	0.633	0.414	-	-
Item13	Classification	72.9	96.4	0.583	0.610	-	-
Item14	Management	83.1	97.3	0.636	0.704	-	-
Item15	Management	85.6	97.1	0.440	0.346	-	-
Item16	Physiology	76.3	96.3	0.331	0.345	-	-
Item17	Physiology	93.2	97.3	0.160	0.346	-	-
Item18	Physiology	72.0	85.0	0.327	0.338	-	+
Item19	Physiology	80.2	96.8	0.442	0.416	-	+
Item20	Classification	77.1	95.7	0.724	0.646	-	-
Item21	Classification	82.2	94.9	0.572	0.596	-	-
Item22	Physiology	91.5	98.5	0.615	0.517	-	-
Item23	Management	87.3	98.5	0.608	0.546	-	-
Item24	Management	88.1	98.5	0.552	0.347	-	-
Item25	Classification	71.2	93.5	0.481	0.451	-	+
Item26	Physiology	60.2	98.5	0.445	0.347	-	-
Item27	Management	93.2	96.9	0.479	0.346	-	-
Item28	Management	66.1	79.0	0.159	0.218	-	*+
Item29	Classification	66.9	91.5	0.543	0.466	-	-
Item30	Management	74.6	98.9	0.723	0.500	-	-

- Non-significant *P*-values

* *P*-values that indicate DIF concerning profession

+ *P*-values that indicate DIF concerning regions

The sensitivity analysis detected a significant difference in mean test scores between obstetricians and first-year residents, between first-year residents and medical students, and between midwives and midwifery students (Table 2), indicating acceptable test discriminating abilities.

**Table 2:**
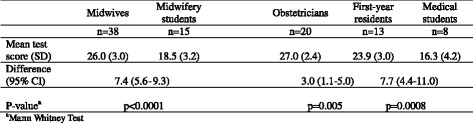
Sensitivity analysis

Mean test scores in the 30-item CTG test for groups with expected differentiated level of CTG knowledge and interpretive skills within each profession (pilot test participants)

**Fig. 3:**
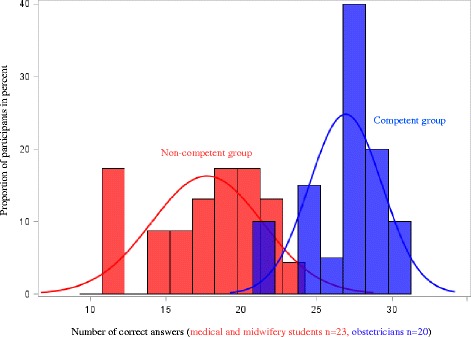
Standard setting in the 30-item CTG test using the *Contrasting Groups method* (pilot test participants)

We decided on a passing score of 25 correct answers, which was found to be the best discriminating point (Fig. 3). The intersection of the two distributions equaled 23, but was adjusted to minimize false-positive errors. The passing score was evaluated on the initial 697 test responses at the CTG courses. A failure rate of 4.6% was detected, which was found to be acceptable by the research group and the *Safe Deliveries* steering committee.

A total of 1801 midwives and physicians participated in the one-day CTG courses. Pilot test participants (*n* = 71) and participants without written consent (*n* = 51) were excluded, thus the included number of participants equaled 1679.

Table 1 presents the 30 items, along with the proportion of correct answers, the fit of the items to loglinear Rasch model, and the results of DIF analyses.

The loglinear Rasch analysis showed a good fit for all items. Evidence of DIF was disclosed in four items related to profession and four items related to regions. No evidence of DIF was disclosed concerning size of maternity unit and seniority. The effect of including and excluding items with DIF are presented and discussed in Additional files 1, 2 and 3.

Many items displayed ceiling effect, which means that a high proportion of the participants answered the item correctly. No floor effect was displayed. Cronbach’s alpha equaled 0.63.

## Discussion

In this validation study, where we aimed to develop a national CTG MCQ test, we found that the process and findings supported our proposed interpretation of the assessment as measuring CTG knowledge, interpretive skills, and clinical decision-making. The learning objectives’ development and item writing, the proofreading and pilot testing, and the sensitivity and Rasch analyses all underpin this. However, in its current form the test does not meet the criteria for a high-stake examination. More items and items with a higher degree of difficulty need to be integrated to increase reliability. In Table 3 we have highlighted the strengths and challenges in the current test development process.

**Table 3:** Strengths and challenges in the test development process

Strengths	
Project group	Consisted of professionals with profound content knowledge, a medical educationalist and a statistician with experience in test-development.
Test content	Based on nationally defined learning objectives, which generated relevant and coverable test content.
Test blueprint	Predefined and based on nationally developed learning objectives.
Test format	MCQ’s, which can test more than simple facts, is suitable for large groups and time- and cost effective. Assess competences at the two lower levels of Millers triangle, *knows* and *knows how*.
Language	Predefined spelling and abbreviations ensured consistency in wordings and terms.
Proofreading	Several proofreaders. Proofreading of content, language and structure/format.
Pilot test participants	A large sample representing in part the intended test-takers.
Pilot testing	Written and verbal feedback gave insight into the pilot participants’ thought processes during testing.
Standard setting	An acknowledged method was used. The passing score was adjusted to minimize false-positive values and was validated on initial test responses.
Psychometric properties	Evaluated on both pilot test responses and the responses from the real test-takers.
Test-takers	A high number of participants enabled the use of advanced statistical analyses such as Rasch analyses.
No. of options in each item	Three or four options were chosen dependent on the numbers of plausible distractors.
Challenges	
Test format	A written assessment cannot assess competences on the two higher levels of Millers triangle, *shows how* and *does* (i.e. clinical performance).
Number of items	More items would expectedly have increased reliability and would have allowed for the development of an item bank.
Item difficulty	Items of a higher difficulty would expectedly have increased reliability and entailed a more challenging test.
Pilot test participants	Medical and midwifery students did not represent the intended test-takers and lowered the percentage of correct answers.
Relations to other variables	There was no test available for comparison.
Context	The context of pilot testing and real testing differed; pilot participants did not attend a one-day teaching course prior to testing and the test was therefore more challenging than in the real setting.
Time devoted for assessment	More items and items with a higher difficulty require more time devoted for assessment in an education program.

The thorough process of learning objectives’ development prior to this study was a robust foundation for the test development process. It generated relevant and coverable test content and a thorough discussion of and clear distinction of the construct of the assessment.

The choice of assessment method and format is always disputable; each has its advantages and disadvantages. Nevertheless, there is general agreement that the content of the test is more important than the response format and MCQ’s can if constructed well, test more than simple facts [11]. A written assessment can, however, only be used to measure certain competences. From the perspective of Miller’s pyramid of competence, the written assessment operates on the two lower levels of competence measurement: *knows* and *knows how* [26]. If the aim is to obtain information about how midwives and physicians perform in a clinical context (*shows how* and *does*), other assessment methods need to be integrated in the education program.

Valuable information was collected in the response process. An item that aimed to measure knowledge about cord blood pH values turned out to be offensive, as the item addressed the neonatal prognosis associated with a low pH value. The item therefore measured ethical considerations rather than knowledge. Another test item that aimed to measure clinical decision-making turned out to be a test of reading because the stem text was too comprehensive. Both items were clearly non-functioning items that required extensive revision or exclusion.

The pilot testing was performed on a large sample representing the intended test-takers, which we perceive a strength of the study. Optimally, we should have performed the pilot testing on participants who had completed the CTG course. This was not possible due to simultaneously development of the test and the CTG course. It implied that sensitivity analyses and standard setting was performed on responses with a lower proportion of correct answers than in the intended context (Table 1). One must be aware that the percentage of correct answers may increase considerably when the test is incorporated in the education program.

When floor or ceiling effect is present the test or the affected items will have poor discrimination ability, as differences are harder to distinguish [24]. The ceiling effect might also have affected the reliability estimate, which was lower than expected in the final test. The fetal monitoring assessments in the United States and Australia contain 100 and 50 items, respectively [7, 23]. Lengthening the CTG test would expectedly result in a higher reliability estimate [9].

Cronbach’s alpha was substantially higher in the pilot test than in the final test, which we believe is attributed both to the inclusion of students among the pilot participants and the above-mentioned lack of course participation among the pilot test participants. This illustrates the importance of context when discussing validity and the importance of choice of pilot test participants.

As literature encourage we strived to set a passing score that was reasonable, defensible and fair [21]. There is no ‘true’ passing score, and all standard-setting methods require judgment and decisions [21]. We find it a strength that the passing score was validated, though we are aware, that this implied a frustrating wait for the course participants.

The large population of CTG course participants and the thorough evaluation of psychometric properties was an additional strength of this study. The fit of the loglinear Rasch model convincingly indicates that the test measures the intended construct. DIF was detected in relation to profession and regions, and the test is therefore not suitable for measuring differences between midwives and physicians or differences across regions. It is not surprising that differences are detected between two professions whose members have different education, competences and responsibilities. As prescribed in patient safety literature [27], it was important for *Safe Deliveries* to function in an interprofessional setting, thereby avoiding the ‘silo approach’ and instead striving for a uniform ‘CTG language’ on a national level. However, as this validation process reveals, it is challenging to develop a uniform test for both professions. An allocation of test items in different levels of competences might be a solution [23].

In *The Standards* internal structure is suggested to be the third validation step, and it was a limitation in our study that the psychometric properties of the test were not examined more thoroughly during the pilot phase. A large amount of test responses are required for Rasch analyses and we therefore chose to evaluate psychometric properties on the actual test-takers.

As demonstrated, the process of test development is complex and time-consuming. Professionals with extensive knowledge of the test content, educationalists, statisticians, time, an implementation plan, economics and stakeholder’s corporation are some of the crucial ingredients in the process.

The question of whether or not to integrate a test in a teaching intervention is disputable. Testing is known to enhance learning [28], it outlines the important topics within a field and it can be a motivating factor for learning. Based on this we believe the current test is an important part of the CTG education program. Certification exams in fetal monitoring has been implemented in obstetric units in USA [29] and a positive effect on clinical outcomes has been suggested [30]. Future studies in Denmark will examine the educational and clinical impact of this national CTG education program. The medical education literature recommends that decisions concerning considerable consequences for individual participants, as a restriction to clinical work at a maternity unit, should not be made based on just one assessment method [9]. Therefore, observational and performance assessments could beneficially be implemented if the test prospectively should function as a high-stake examination.

One of the considerable overall challenges in developing a CTG test are the well-known limitations of the surveillance method; Nonetheless, electronic fetal monitoring is widely integrated in the care and management of labor, which makes development and maintenance of competences crucial.

## Conclusions

Test development is complex and time-consuming, and the importance of context cannot be overemphasized. The five-step unitary validation approach was a useful framework for the development of a CTG MCQ test. Our process and findings support the proposed inferences of the test, but a higher reliability is needed for the CTG test to function as a high-stake assessment. This study provides a feasible template relevant for MCQ test development in general. Applying the unitary approach to validity will expectedly lead to improved assessments in medical education.

## Additional files

Additional file 1: Supplementary details on psychometric properties and the statistical aspects of validation. (DOC 39 kb)

Additional file 2: The magnitude of differential item functioning (DIF) with respect to profession. Proportion of correct answers for item 1, 7, 8 and 28 for physicians and midwives with equal amount of correct answers in remaining items. (PDF 96 kb)

Additional file 3: The impact of differential item functioning (DIF). Proportion of correct answers among physicians and midwives in hypothetical sub-tests formed by including or excluding items with DIF. (DOC 33 kb)

## Abbreviations

CTG: Cardiotocography
DIF: Differential item functioning
MCQ: Multiple-choice question
