# Supplementary material for: Development of a written assessment for a national interprofessional cardiotocography education program
Source: BMC Med Educ. 2017 May 18;17:88. doi: 10.1186/s12909-017-0915-2 (PMC5437628; doi:10.1186/s12909-017-0915-2)
Supplement: Supplementary file 1 — Supplementary details on psychometric properties and the statistical aspects of validation. (DOC 39 kb) [file 12909_2017_915_MOESM1_ESM.doc]

Additional file 1. Supplementary details on psychometric properties and the statistical aspects of validation

**Development of a written assessment for a national interprofessional cardiotocography education program**

Line Thellesen, Thomas Bergholt, Morten Hedegaard, Nina Palmgren Colov, Karl Bang Christensen, Kristine Sylvan Andersen, Jette Led Sorensen

The degree of validity of the intended inferences of the test results can be studied by looking at the fit of the data to a psychometric model. We used the Rasch model [1,2] and an extension of that model, the loglinear Rasch model [3,4]. We evaluated the fit of the individual items using an item fit statistic [5] that evaluates the observed correlation between an item and the sum of the remaining items.

The Rasch model imposes measurement requirements on the data and can be seen as a mathematical formulation of ideal measurement requirements [4]. Some of these requirements are technical, while others are essential. An example of the former is the requirement of local independence, which means that the underlying latent variable (in the current test: CTG knowledge, interpretive skills and clinical decision-making) explains all the correlation between any pair of items. An example of an essential requirement is that the difficulty of an item does not depend on external variables such as the profession or the seniority of the respondent.

Local independence is the underlying assumption of latent variable models. The observed items are conditionally independent of each other, given an individual score on the latent variable(s). This means that the latent variable explains why the observed items are related to other items [6]. This requirement, called local independence, is unrealistic for the current test because some items share a common stem and others share a common topic. The loglinear Rasch model is an extension in which local dependence can be added.

In the first analysis, the Rasch model rejected 10 of the 30 items (results not shown) and a loglinear Rasch model was used instead. We added local dependence for four item pairs (items 2 and 3, items 13 and 14, items 20 and 21, and items 22 and 23) as they shared a common stem. In this extended model only five out of the 30 items were rejected. We also found evidence of local dependence for three item pairs (items 2 and 25, items 3 and 4, and items 3 and 29) that covered CTG classification and for items 12 and 19 that covered fetal physiology. In this model three items were rejected. Adding local dependence for a single additional item pair (items 9 and 28) yielded a model where no strong evidence of item misfit was disclosed. In this model, only three items (items 9, 12, and 17) were significant at the 5% level. Adjusting for multiple testing, using the Benjamini and Hochberg procedure [7] to control the false discovery rate, indicated that these were type I errors.

Differential item functioning (DIF) occurs when respondents from different groups, such as people from different professions with the same ability, have a different probability of responding correctly to an item in a test [8]. An item does not display DIF if people from different groups have a different probability of giving a correct response; it only displays DIF if people from different groups with the same underlying true ability have a different probability of giving a correct response.

When testing for DIF we found that items 1, 7, 8, and 28 functioned differently for physicians and midwives and that items 18, 19, 25, and 28 functioned differently across regions. No evidence of DIF was disclosed concerning seniority or size of maternity unit. The psychometric properties of the test are summarized in Table 1.

To study the magnitude of DIF we computed, for each item revealing DIF, the proportion of physicians and midwives, respectively, who gave a correct answer (Additional file 2). For items 1, 7, 8, and 28, midwives consistently had a higher probability of giving a correct answer than physicians with the same score on the remaining items. Thus, including items that function differently will lead to different comparisons of physicians and midwives. Additional file 3 illustrates this, showing group comparisons based on three different sub-tests: (i) the total 30-item test, (ii) a reduced 26-item test in which the DIF items favoring midwives are removed, and (iii) a reduced four-item test with the items favoring midwives. The former two show no significant difference, whereas the latter shows significantly different group means.

References

1. Rasch G. Probabilistic Models for Some Intelligence and Attainment Tests. Danish National Institute for Educational Research, Copenhagen, 1960.

2. Fischer GH, Molenaar IW. Rasch models: Foundations, recent developments, and applications. Springer-Verlag, New York, 1995.

3. Kelderman H. Loglinear Rasch model tests. Psychometrika.1984;49(2):223–45.

4. Kreiner S, Christensen KB. Validity and objectivity in health-related summated scales: analysis by graphical loglinear Rasch models. In Von Davier M, Carstensen CH Multivariate and mixture distribution Rasch Models: Extensions and Applications. Spinger-Verlag, New York, 2007.

5. Kreiner S. A Note on Item-Restscore Association in Rasch Models. Applied Psychological Measurement. 2011; 35(7):557–61.

6. Lazarsfeld PF, Henry NW, Anderson TW. Latent structure analysis. Houghton Mill, Boston,1968.

7. Benjamini Y, Hochberg Y. Controlling the false discovery rate: A practical and powerful approach to multiple testing. Journal of the royal statistical society. 1995;57:289–300.

8. Holland PW, Wainer H. Differential Item Functioning. Hillsdale, NJ: Lawrence Erlbaum, 1993.
