# Supplementary figures and images for: Development of a written assessment for a national interprofessional cardiotocography education program
Source: BMC Med Educ. 2017 May 18;17:88. doi: 10.1186/s12909-017-0915-2 (PMC5437628; doi:10.1186/s12909-017-0915-2)

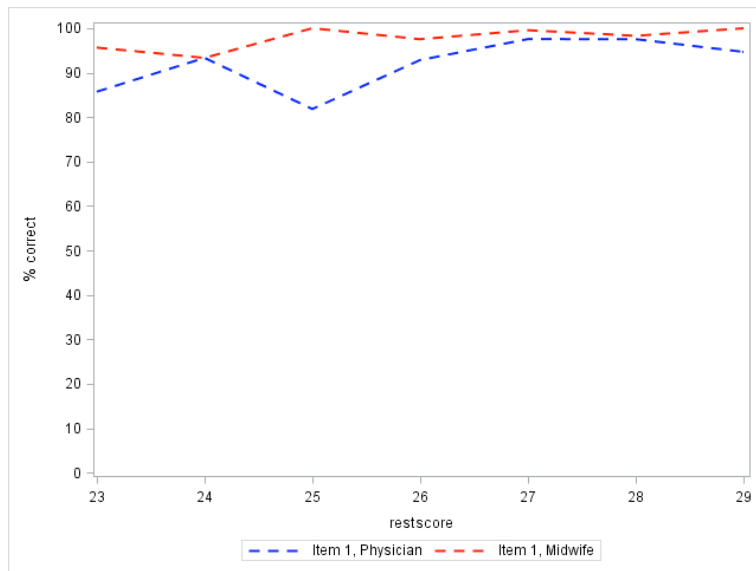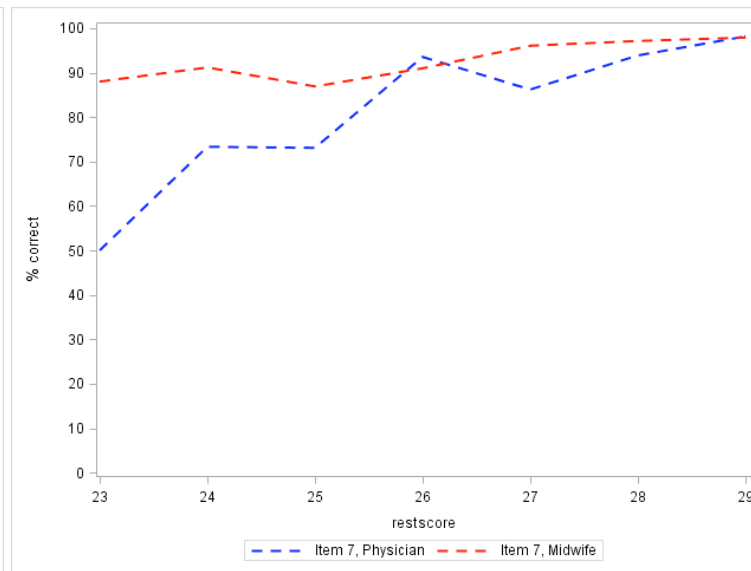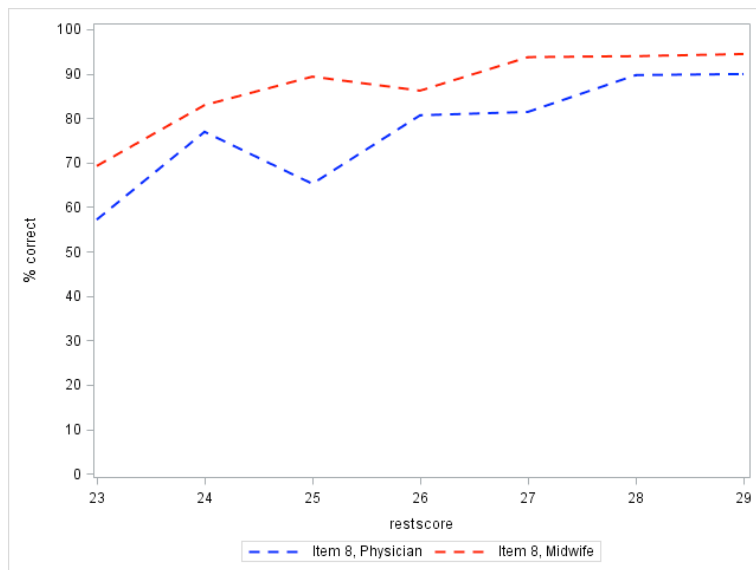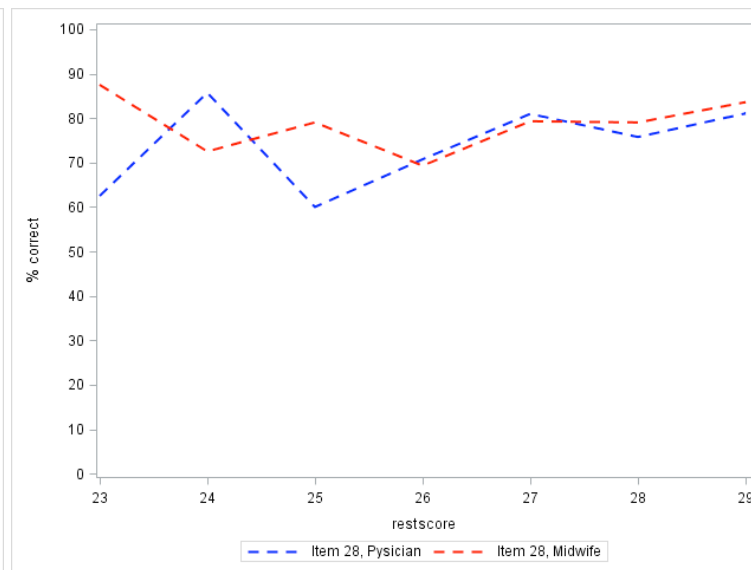

Supplement: Supplementary file 2 — The magnitude of differential item functioning (DIF) with respect to profession. Proportion of correct answers for item 1, 7, 8 and 28 for physicians and midwives with equal amount of correct answers in remaining items. (PDF 96 kb) [file 12909_2017_915_MOESM2_ESM.pdf]
