# Supplementary material for: Development of a written assessment for a national interprofessional cardiotocography education program
Source: BMC Med Educ. 2017 May 18;17:88. doi: 10.1186/s12909-017-0915-2 (PMC5437628; doi:10.1186/s12909-017-0915-2)
Supplement: Supplementary file 3 — The impact of differential item functioning (DIF). Proportion of correct answers among physicians and midwives in hypothetical sub-tests formed by including or excluding items with DIF. (DOC 33 kb) [file 12909_2017_915_MOESM3_ESM.doc]

| Number of items | Proportion of correct answers | | | | Group comparison | |
| --- | --- | --- | --- | --- | --- | --- |
|  | Physician | | Midwife | |
|  | mean | (SD) | mean | (SD) | Difference | P-value* |
| 30 items | 0.94 | (0.06) | 0.94 | (0.07) | 0.00 | 0.63 |
| 26 items^1^ | 0.95 | (0.05) | 0.95 | (0.07) | 0.01 | 0.19 |
| 4 items^2^ | 0.87 | (0.17) | 0.91 | (0.15) | -0.04 | 0.00 |
|  |  |  |  |  |  |  |

*^1^ Items 1, 7, 8 and 28 excluded*

*^2^ Items 1, 7, 8 and 28*

** Mann Whitney test*
